# Preeclampsia as a Study Model for Aging: The Klotho Gene Paradigm

**DOI:** 10.3390/ijms26030902

**Published:** 2025-01-22

**Authors:** Monia Cecati, Stefania Fumarola, Salvatore Vaiasicca, Laura Cianfruglia, Arianna Vignini, Stefano Raffaele Giannubilo, Monica Emanuelli, Andrea Ciavattini

**Affiliations:** 1 Department of Human Sciences and Promotion of the Quality of Life, San Raffaele Roma Open University, 00166 Rome, Italy; monia.cecati@uniroma5.it; 2 Scientific Direction, IRCCS INRCA, 60124 Ancona, Italy; s.fumarola@inrca.it (S.F.); s.vaiasicca@inrca.it (S.V.); l.cianfruglia@inrca.it (L.C.); 3 Department of Clinical Sciences, Section of Biochemistry, Biology and Physics, Università Politecnica Delle Marche, 60126 Ancona, Italy; m.emanuelli@univpm.it; 4 Department of Clinical Sciences, Clinic of Obstetrics and Gynaecology, Università Politecnica Delle Marche, 60123 Ancona, Italy; a.ciavattini@univpm.it

**Keywords:** preeclampsia, aging, age-related disorders, Klotho, epigenetics

## Abstract

Aging and pregnancy are often considered opposites in a woman’s biological timeline. Aging is defined by a gradual decline in the functional capabilities of an organism over its lifetime, while pregnancy is characterized by the presence of the transient placenta, which fosters the cellular fitness necessary to support fetal growth. However, in the context of preeclampsia, pregnancy and aging share common hallmarks, including clinical complications, altered cellular phenotypes, and heightened oxidative stress. Furthermore, women with pregnancies complicated by preeclampsia tend to experience age-related disorders earlier than those with healthy pregnancies. Klotho, a gene discovered fortuitously in 1997 by researchers studying aging mechanisms, is primarily expressed in the kidneys but also to a lesser extent in several other tissues, including the placenta. The Klotho protein is a membrane-bound protein that, upon cleavage by ADAM10/17, is released into the circulation as soluble Klotho (sKlotho) where it plays a role in modulating oxidative stress. This review focuses on the involvement of sKlotho in the development of preeclampsia and age-related disorders, as well as the expression of the recently discovered Mytho gene, which has been associated with skeletal muscle atrophy.

## 1. Introduction

Preeclampsia is a placenta-mediated, multisystem hypertensive disorder of pregnancy that affects 2–8% of pregnancies [1]. In its severe form, it affects about 1.4% of pregnancies in the Western world, is characterized by maternal organ damage and/or fetal growth restriction, and is a major cause of maternal and infant morbidity and mortality [2,3].

One year after pregnancy, women have higher cardiovascular risks, such as hypertension, obesity, and dyslipidemia, making them 2–7 times more likely to develop cardiovascular diseases (CVDs) early compared to those with a normotensive pregnancy [4,5]. Women with preeclampsia defined as “early” preeclampsia—that is, at onset before 34 weeks’ gestation—have an even higher risk of up to 9 times higher risk of death from cardiovascular disease [6]. Preeclampsia is characterized by increased oxidative stress, vascular damage, and endothelial and metabolic dysfunctions [7,8,9,10,11].

Oxidative stress can activate several transcription factors, which lead to the expression of genes involved in the activation of inflammatory pathways favoring the production of inflammatory cytokines and the establishment of an inflammatory environment, which is involved in the onset and progression of several diseases [12,13,14,15,16,17,18].

The increased oxidative stress, due to placental hypoxia, is also involved in the chronic inflammatory state characterizing preeclamptic pregnancies [19]. Although preeclampsia and cardiovascular disease share common risk factors such as hypertension and obesity [20], there is evidence that preeclampsia is accompanied by vascular biochemical dysfunction that lingers throughout a woman’s life and, in later life, manifests itself as a potentially life-threatening disease [21,22]. Many studies in recent years have hypothesized and demonstrated genetic variants that may influence the risk of developing preeclampsia [23]. The heritability of preeclampsia is estimated at about 55% [24,25]. Genome-wide association studies (GWASs) [26] have reported maternal and fetal heritability for Europe as 38.1% and 21.3%, respectively, while those for Central Asia have reported 54.4% and 42.5%, respectively. In addition to the maternal association, it is also necessary to consider the fetal/neonatal association to future cardiovascular disease. The incidence of cardiovascular morbidity is significantly higher among infants born to preeclamptic women [27]. The pathophysiological basis of preeclampsia in fact lies in the altered invasion of the trophoblast and remodeling of the spiral artery, resulting in the restriction of uteroplacental circulation [28,29]. The fetus of a preeclamptic woman is in a setting of multiple insults, including ischemia, hypoxia, placental insufficiency, imbalance of angiogenic factors, inflammation, and oxidative stress [30]. The fetuses of preeclamptic women exhibit microcirculation abnormalities, endothelial disfunction, and accelerated progression of atherosclerosis [31]. The mechanisms connecting in utero exposure to preeclampsia with an increased risk of future cardiovascular disease may involve a complex interaction between shared genetic factors, environmental influences, and developmental programming. CVDs are also closely linked to the aging process. Specifically, in elderly women (>60 years) the prevalence of CVDs is even higher than in men [32].

At first glance, pregnancy and aging may appear to be opposing processes: pregnancy is characterized by the presence of the transient placenta, which develops the cellular fitness required to support fetal growth, while aging is marked by a progressive decline in cellular function. However, when considering preeclampsia, both pregnancy and aging may share common cellular hallmarks, which will be explored further in this paper.

## 2. Methods

To identify relevant studies, we conducted a comprehensive literature search across several databases, including PubMed, Scopus, and Web of Science. The search aimed to locate significant English-language research articles and reviews published in peer-reviewed journals over the past two decades (2000–2024). However, we also included foundational studies on preeclampsia and the Klotho gene published before this period. Our focus was on both in vivo human and animal studies to ensure clinical relevance while excluding studies lacking significant outcomes related to preeclamptic pregnancies or age-related disorders.

The search terms were carefully selected to encompass a wide range of research on the relationship between Klotho expression and preeclampsia or aging. The following keywords and phrases, in various combinations, were used: ‘Klotho’, ‘Healthy pregnancies’, ‘Preeclamptic pregnancies’, ‘Age-related disorders’, ‘Epigenetic modifications’, ‘Klotho expression in pregnancies’, ‘Klotho dysregulation in preeclamptic pregnancies’, and ‘Klotho dysregulation in age-related disorders’. Boolean operators (AND, OR, NOT) were applied to refine and expand the search.

Inclusion criteria focused on studies exploring the effects of Klotho administration in cell cultures or animal models, as well as research on peripheral Klotho concentrations or tissue expression in both young and elderly humans. Exclusion criteria ruled out non-English studies, articles published before 1994, and those lacking relevant clinical or experimental data. This approach ensured a thorough and wide-reaching review, emphasizing studies that provided clinical insights and a mechanistic understanding of the relationship between Klotho, preeclampsia, and age-related disorders.

## 3. Preeclampsia, Aging and Cellular Phenotype

Aging is characterized by a gradual decline in the functional capabilities of an organism over the course of its life. During the aging process, multiple organs undergo progressive deterioration of their physiological functions, leading to the onset of various diseases [33].

To date, impaired angiogenesis [34] and inflammation [35] are the two mechanisms known to be the most responsible for the pathophysiology of preeclampsia. The poor trophoblast invasion of the spiral arteries [28,36,37] in the placenta bed compromises placentation [38] and vascularization [39], induces an inappropriate activation of the immune system [40], and results in an inevitable dysregulation of several metabolic pathways [41,42]. The inability of preeclamptic tissue to return to a physiological state initiates an accelerated aging process, driving the placenta toward premature senescence and impairing its ability to sustain fetal growth until the normal conclusion of pregnancy. Cellular senescence is one of the aging hallmarks. Senescent cells were discovered by Hayflick and Moorhead in 1961 [43] and were later defined as cells that exit the cell cycle and enter replicative senescence due to the loss of their proliferative potential [44]. Telomere shortening is a hallmark of cellular senescence. In humans, telomeres consist of tandem repeats of the DNA sequence (TTAGGG) at the ends of chromosomes. These telomeres play a crucial role in maintaining chromosome stability and protecting coding DNA from recombination, degradation, and replication damage, a function recognized for many years.

In adult human cells, the reduced activity of DNA telomerase leads to telomere shortening. With each cell division, telomeres become progressively shorter. To prevent telomere length from falling below a critical threshold, a permanent cell cycle arrest occurs. Shortened telomeres are associated with age-related diseases, including metabolic disorders (e.g., metabolic syndrome, liver cirrhosis) and cardiovascular conditions (e.g., atherosclerosis) [45,46].

Telomeres in preeclamptic trophoblast tissue are shorter compared to those in physiological trophoblast tissue [47,48]. In preeclampsia, excessive fetal hypoxia during the early stages of placentation induces DNA damage in the telomeric region. This dysfunctional telomere length, combined with reduced telomerase activity, may contribute to the accelerated placental aging observed in preeclamptic pregnancies [49].

Telomerase activity and the maintenance of telomere length are known to be linked to the immortality of cancer cells, germline cells, and embryonic stem cells [50]. The expression of genes related to undifferentiated embryonic stem cells is increasingly being used as a biomarker for tissue aging in a growing number of studies [51,52,53]. A reduced expression of stem cell markers is reported in trophoblast tissue from preeclamptic pregnancy [47] but also in aging tissue as synonyms of decline in tissue regenerative potential [54].

Although quiescent in the cell cycle, senescent cells undergo significant changes in their gene expression profile. Specifically, they are characterized by the secretion of cytokines, chemokines, and proteases with pro-inflammatory properties, a phenomenon known as the senescence-associated secretory phenotype (SASP) [55].

Strong evidence indicates that the senescence-associated secretory phenotype (SASP) is closely associated with critical clinical parameters and outcomes, with its impact intensifying with age. This is likely a result of the growing accumulation of senescent cells in one or more organs as biological age progresses [56]. Published studies also highlight dysregulated levels of pro-inflammatory cytokines associated with SASP in older individuals.

The literature reports age-related increments of IL-6 [57], IL-8 [58], monocyte chemoattractant protein 1 (MCP-1) [55], and plasminogen activator inhibitor 1 (PAI-1) [59] in blood concentrations from elderly women. Additionally, IL-6 levels in venous blood from older women (over 65 years of age) are positively correlated with high blood pressure and negatively correlated with estimated glomerular filtration rate (eGFR), a test used to assess kidney function and determine the stage of kidney disease [60]. Notably, serum IL-8 levels were significantly increased in patients affected by chronic liver disease, with respect to healthy subjects [61]. Duisenbek et al. reported that MCP-1 levels were higher in the blood of elderly patients affected by type 2 diabetes and cardiovascular disease than that of healthy subjects [62].

Stubblefield et al. demonstrated that PAI-1 was significantly increased in patients affected by type 2 diabetes and metabolic syndrome when compared with disease-negative controls [63]. Type 2 diabetes, cardiovascular diseases, and kidney and liver failure are clinical hallmarks of both aging and preeclampsia. In a study by Suvakov et al., patients with pregnancies complicated by preeclampsia were recruited. They demonstrated that women with preeclampsia had elevated levels of inflammatory mediators (e.g., IL-6, IL-8, MCP-1, and PAI-1) in both blood and adipose tissue compared to women with healthy pregnancies [64].

Aging also impacts mitochondrial function, diminishing the efficiency of oxidative phosphorylation. This decline is linked to altered ATP production and an increase in the generation of reactive oxygen species (ROS) [65]. Excessive ROS can lead to the oxidation of mitochondrial DNA, proteins, and lipids, impairing the cell’s ability to activate mitophagy and ultimately resulting in mitochondrial dysfunction [66,67].

Studies have shown that mitochondrial dysfunction in elderly women is associated with cardiovascular disease [68], increased risk of cardiovascular disease [69], and metabolic syndrome [70].

The content of extracellular vesicles released into the circulation in mitochondrial DNA (mtDNA) was higher in frail elderly women compared to healthy subjects [71].

Since the late 1980s, mitochondrial disorders have been identified as potential contributors to preeclampsia [72,73]. Prolonged hypoxia or recurrent cycles of ischemia/reoxygenation during preeclampsia can further exacerbate mitochondrial dysfunction [74]. Serum levels of mtDNA have been shown to be elevated in pregnancies complicated by preeclampsia compared to healthy pregnancies [75].

mtDNA is not the only circulating biomarker associated with aging and preeclampsia. Circulating microRNAs (miRNAs) also play a significant role in the pathogenesis, diagnosis, and prediction of both preeclampsia and aging. Given their potential therapeutic implications for treating preeclampsia and age-related diseases, the following section will focus on miRNAs, as well as on two other key epigenetic modifications: DNA methylation and histone modification.

## 4. Preeclampsia, Aging, and Epigenetics

The term “epigenetics” was first introduced in the early 1940s, and its meaning has evolved significantly since Conrad Waddington’s definition in 1968 [76]. Today, epigenetics is generally understood as “the study of changes in gene function that are mitotically and/or meiotically heritable and that do not involve a change in the DNA sequence” [77]. The three most studied epigenetic modifications are DNA methylation, microRNAs (miRNAs), and histone modification. Abnormal epigenetic modifications to DNA have been observed in cells from various sources, not only in elderly individuals but also in pregnant women with preeclampsia. In this section, we aim to provide a concise overview of the existing literature, focusing on epigenetic modifications in peripheral blood cells to highlight the similarities between aging and preeclampsia. DNA methylation is a heritable epigenetic modification that involves the covalent transfer of a methyl group to the C-5 position of the cytosine ring in DNA [78].

This reaction is catalyzed by DNA methyltransferases (DNMTs), which use S-adenosylmethionine (SAM) as the methyl donor. SAM, however, also serves as a co-substrate for many other methyltransferases [79]. DNA methylation is mediated by a family of DNMTs: DNMT1, DNMT2, and the maintenance DNMTs, along with DNMT3A, DNMT3B, and DNMT3L, which are referred to as de novo DNMTs [80,81].

In somatic cells, more than 98% of DNA methylation occurs in CpG-rich regions of the genome [80]. DNMT1 and DNMT2 primarily methylate hemimethylated DNA in vitro and are active during the S phase of replication. In contrast, DNMT3A, DNMT3B, and DNMT3L prefer unmethylated CpG dinucleotides and are involved in de novo methylation during development [80,81].

In 2014, White et al. demonstrated that maternal leukocyte DNA methylation was altered in pregnancies with preeclampsia compared to healthy pregnancies. Specifically, differential methylation of certain neuronal genes, including GRIN2b, GABRA1, PCDHB7, and BEX1, was associated with an increased risk of preeclampsia [82]. In 2016, Rahat et al. studied the methylation level in the STAT5A promoter region by recovering cell-free fetal DNA from maternal plasma in both preeclamptic and healthy pregnancies. The study showed that hypermethylation of the STAT5A promoter region was positively correlated with the onset of preeclampsia [83]. Additionally, Zakeri et al. used the methylation-specific PCR method to identify DNA hypermethylation in the promoter region of the Nrf2 gene in the peripheral blood of preeclamptic individuals [84]. Hypermethylation in the promoter regions of both p21 and TP53 genes was also linked to preeclampsia [85].

Weiping et al. demonstrated that preeclampsia is associated with reduced methylation levels in the promoter region of the GNA12 gene. Furthermore, the methylation level of the GNA12 promoter was found to be similar in both the placenta and peripheral blood of pregnant women [86]. Lu et al. also identified an association between preeclampsia and hypomethylation in the promoter region of the CTGF gene, observed in both placentas and peripheral blood from a Chinese population [87]. Maternal vascular dysfunction, a hallmark of preeclampsia, has been linked to DNA methylation changes. Mousa et al. and Walsh et al. demonstrated that the methylation levels in the promoter regions of TBXAS1 [88], MMP-1 [89], and IL-17 cytokines [90] were reduced in the systemic blood vessels of preeclamptic women. Several of the genes discussed above also exhibit similar methylation patterns in age-related diseases. Hypermethylation in the promoter of the p21 gene has been strongly correlated with acute lymphoblastic leukemia and poor prognosis in elderly women [91]. In patients with type 2 diabetes mellitus, with or without nephropathy, methylation levels in the promoter region of the CTGF gene were significantly lower compared to the control group [92]. Additionally, a significantly decreased methylation level in the IL-17 promoter was observed in patients with age-related macular degeneration [93].

Aberrant DNA methylation (hypermethylation or hypomethylation) at promoter CpG islands leads to transcriptional silencing or the overexpression of genes and miRNAs. This disruption can cause the improper execution of key cellular pathways, much like genetic changes (e.g., mutations or deletions) [94].

miRNAs are single-stranded, non-coding RNAs, typically 20–24 nucleotides in length, derived from the primary miRNA transcript (pri-miRNA) [95]. The pri-miRNA is enzymatically processed, and the resulting mature miRNA is incorporated into the RNA-induced silencing complex (RISC) with the Argonaute2 protein, which induces mRNA target silencing [96,97]. Once released from the cell, often within extracellular vesicles such as exosomes and microvesicles [98,99], miRNAs exert their suppressive function by targeting mRNA, reaching recipient cells through peripheral circulation [100,101]. Due to their abundant presence in all body fluids, miRNAs serve as valuable biomarkers for a variety of diseases, including preeclampsia and aging [102,103]. Wang et al. isolated circulating exosomes from the peripheral blood of preeclamptic women and found an upregulation of miR-15a-5p compared to healthy pregnant women [104]. Circulating miR-132 [105] and miR-151a-3p [106] were significantly higher in preeclamptic women than in controls. A decrease in miR-93-5p and miR-126-3p levels in peripheral circulation, when associated with the overexpression of miR-125, increased the risk of preeclampsia. miR-125 inhibits cytotrophoblast invasion and impairs endothelial cell function [104]. The apoptosis-related miR-21 was overexpressed in the maternal plasma of women with preeclampsia and has been used as a biomarker to distinguish between mild and severe preeclampsia [107].

miR-15a-5p [108] and miR-151a-3p [109] were also found to be upregulated in the peripheral blood of elderly women with cognitive decline. Elderly women with mild cognitive impairment showed an increased expression of miR-132 compared to age-matched controls [110]. Kocijan et al. investigated the circulating miRNA profile in the peripheral blood of patients with idiopathic and postmenopausal osteoporosis and fragility fractures, suggesting miR-93-5p as a potential biomarker for bone-related pathologies [111]. Circulating miR-126-3p levels were lower in the peripheral blood of 246 hospitalized geriatric patients with cardiovascular diseases and multimorbidity and were positively associated with a higher risk of short- and medium-term mortality [112].

Olivieri et al. conducted a miRNA plasma profiling study with 11 healthy individuals aged 20, 80, and 100 years. They validated the selected miRNAs in a cohort of 111 healthy adults aged 20–105 years and 30 centenarians. Additionally, 34 patients with CVDs and 15 healthy centenarian offspring (CO) were included in the study. The results showed that miR-21 expression was higher in CVD patients and lower in CO compared to age-matched controls [113].

Histones, the protein components of chromatin, undergo posttranslational modifications that influence chromatin structure [114]. Histone acetylation and histone methylation are the two most widely studied modifications in the context of preeclampsia pathology. Histone acetylation is catalyzed by histone acetyltransferases and leads to gene transcriptional activation [115] by making the DNA–protein complex more accessible to transcription factors and RNA polymerase II [116].

Unlike acetylation, the methylation of lysine residues can have varying effects on gene expression, depending on the specific lysine residue that undergoes methylation. Among the identified methylated lysine residues, several have been extensively studied. For example, methylation at lysine 4 (K4) of histone 3 (H3) promotes gene expression, while methylation at K9 of H3 is linked to transcriptional repression [117].

Whole genome analysis of cytotrophoblasts from severe preeclampsia revealed a significant increase in H3K27 acetylation. Interestingly, genes that are typically downregulated during a physiological pregnancy at term were found to be upregulated in this syndrome [118].

When analyzing the expression of histone proteins H3K4me3 and H3K9ac in both preeclamptic and control placentas through immunohistochemistry, Meister et al. observed a significant global reduction in trimethylated histone H3K4me3 and acetylated H3K9ac in preeclamptic tissue. These findings suggested a loss of accessibility for genes to be transcribed [118,119]. Sheng et al. specifically evaluated histone H3K9 methylation protein expression in HUVECs from normal and preeclamptic pregnancies. Their data showed a strong correlation between di- and tri-H3K9 methylation and reduced antioxidant CuZn-SOD expression in fetal endothelial cells from preeclampsia, reinforcing the critical role of oxidative stress in the development of this syndrome [120].

Histone methylation is closely related to oxidative stress. Whongsiri et al. demonstrated that in bladder cancer tissues, an increase in 8-OHdG was associated with elevated tri-methylated H3K9, supporting the hypothesis that oxidative stress also influences genome reprogramming [121]. Aging has also been linked to defective muscle regeneration due to a reduction in H3K9 di/tri-methylation [122]. In esophageal squamous cell carcinoma, Chen et al. observed the global hypoacetylation of H3 and H4, along with hypermethylation of H3K4 and H3K27. Furthermore, these modifications were correlated with the severity of the disease [123]. Oxidative stress plays a pivotal role in regulating the system of epigenetic modifications and contributes to the pathophysiology of age-related disorders and preeclampsia, as discussed by the authors [124,125].

## 5. Oxidative Stress in Preeclampsia and Aging

Oxidative stress results from an imbalance between reactive oxygen species (ROS) and antioxidants. ROS are primarily oxygen-based molecules, including the superoxide anion radical (O_2_^−^), hydrogen peroxide (H_2_O_2_), and hydroxyl radicals [126]. These reactive species can structurally damage cellular compounds such as DNA, lipids, carbohydrates, and proteins, necessitating a balance with antioxidant defenses [126,127,128,129]. Under physiological conditions, ROS function as signaling molecules [130,131] playing a role in mediating maternal immune tolerance to the semi-allogenic fetus [132], similar to the role of adenosine [133].

Oxidative stress is implicated in the onset and progression of various cancerous and non-cancerous diseases [134,135,136,137,138].

It also regulates key signaling networks, including forkhead transcription factors of the O class (FoxO) [139]. During a healthy pregnancy, the development of fetal organs and tissues leads to increased metabolic activity and oxygen consumption. Fatty acids are utilized to produce energy for sustaining maternal retroplacental tissues. This process inevitably generates ROS, particularly H_2_O_2_. However, in a physiological pregnancy, oxidative stress triggers antioxidant mechanisms that modulate enzyme activity and interact with non-enzymatic free radical deactivators, helping to eliminate excess ROS and maintain cellular homeostasis [140]. Pregnancy is a state where the balance between ROS and antioxidant defenses can be easily disrupted. Under pathological conditions, elevated ROS levels can stimulate pathological redox signaling, leading to cellular damage and contributing to various disease states [141,142]. Studies focused on pregnancy have shown that oxidative stress is associated with complications during pregnancy and may impact fetal development [143]. Preeclampsia is a pregnancy-related pathology linked to oxidative stress. Although the exact etiology of preeclampsia remains unclear, the underlying pathological event appears to be endothelial injury, which results from elevated placental ROS levels or reduced antioxidant activity [144,145]. This injury leads to hypoxia, which triggers the conversion of xanthine dehydrogenase to xanthine oxidase, further increasing ROS production in the placenta [146,147]. Preeclamptic placentas are also characterized by mitochondrial abnormalities and disruptions in the molecular pathways regulating mitochondrial function [148].

Aging is closely linked to oxidative stress [149], as the increased production of ROS during aging is primarily attributed to mitochondrial dysfunction. This creates a vicious cycle, where oxidative stress exacerbates mitochondrial dysfunction [150]. Oxidative stress plays a significant role in the age-related decline of organ function, including kidney function. Chronic kidney disease (CKD) becomes more prevalent with age, affecting 27.9% of individuals over 70, compared to only 8.1% in the general population [151]. Kidney tubular cells are particularly sensitive to oxidative stress, adopting a senescent phenotype [152]. Autophagy is a key mechanism by which proximal tubular cells maintain homeostasis and protect the kidneys from injury. Yamamoto et al. observed that inadequate autophagic flux in response to metabolic stress increases the risk of age-related kidney diseases [153]. Oxidative stress and imbalanced ROS levels are also implicated in the development of Alzheimer’s disease in aging brains. Martin-Maestro et al. studied mitophagy in hippocampal samples from Alzheimer’s patients, finding that alterations in the mitophagy process were strongly associated with increased ROS production and clinical manifestations of the disease [154]. In human lung epithelial A549 cells, exposure to a high phosphate medium resulted in elevated ROS levels, and the inhibition of antioxidant pathways led to apoptosis of the A549 cells [155].

## 6. The Klotho Gene

In 1997, while exploring the human genome, a research group led by Kuro-o discovered a gene by chance. As their focus was on aging mechanisms, Kuro-o decided to name the gene Klotho, after the Greek goddess Clotho, who spins the thread of life [156]. The Klotho gene in humans is located on chromosome 13q12 and spans over 50 kb.

Subsequently, the name α-Klotho was introduced to refer to the “original” Klotho gene in humans, distinguishing it from its homolog in mice, which was named β-Klotho [157]. In humans, β-Klotho shares 41% amino acid sequence similarity with α-Klotho and is predominantly expressed in adipose tissue and the liver and pancreas. Functionally, β-Klotho acts as a cofactor for fibroblast growth factor 21 (FGF21), an endocrine factor synthesized in the liver that stimulates glucose uptake in adipocytes [158]. In this paper, the term Klotho will refer specifically to α-Klotho.

The human Klotho gene consists of five exons, which, through alternative RNA splicing, generate two distinct transcripts that encode either a membrane-bound or secreted protein. The first transcript is 3036 bp long (encoding 1012 amino acids) and encodes a single-pass membrane protein. This protein includes an N-terminal signal sequence, a putative extracellular domain with two internal repeats (KL1 and KL2, which share 21% similarity to each other), a single membrane-spanning region, and a short intracellular domain. The membrane protein can undergo enzymatic cleavage by ADAMTS 10/17, two members of the disintegrin and metalloprotease family, releasing the entire extracellular domain into the circulation [159,160].

The second transcript of Klotho includes the full 3036 bp encoding the membrane form, with an additional 50 bp insertion, bringing the total length to 3086 bp. However, this insertion contains a premature stop codon, which causes a block in the cDNA translation process, resulting in the synthesis of a truncated protein of 549 amino acids. This truncated protein retains the KL1 domain but lacks the second internal repeat of the extracellular domain (KL2), the transmembrane domain, and the intracellular domain. Consequently, this truncated protein is thought to be secreted outside the cell [161].

The existence of a secreted Klotho protein arising from this second transcript has not been definitively demonstrated. Moreover, recent advances in RNA surveillance suggest that the premature termination codons in the alternative Klotho mRNA (encoding the secreted form of Klotho) leads to mRNA degradation via nonsense-mediated decay (NMD) [162].

As a result, when the authors refer to the soluble form of Klotho (sKlotho) in this review, they are specifically referring to the form generated from the membrane protein (mKlotho) after enzymatic cleavage. mKlotho is predominantly expressed in normal kidneys, but also to a lesser extent in the brain, parathyroid glands, peripheral blood cells, vascular tissue, and placenta [47,163].

Interestingly, sKlotho is more abundant than mKlotho in several tissues, including the kidney, prostate, brain, hippocampus, placenta, and small intestine [161]. sKlotho is found in blood, urine, and cerebrospinal fluid [164] where it exerts cytoprotective effects on various organs, including the kidney. In the following section, the authors will discuss the protective roles of Klotho in the kidney, lung, brain, liver, and peripheral blood vessels (including cardiovascular diseases), which are key organs affected by dysfunctional disorders in age-related diseases and preeclamptic pregnancy.

### 6.1. Klotho Gene and Cytoprotective Effects

Due to its antioxidative properties, Klotho has been linked to several biological pathways and mechanisms. The first study investigating this property was published by Nagai et al. in 2003 [165] where the authors examined the role of oxidative stress in Klotho mutant mice. They focused on neurochemical and behavioral changes associated with aging and found that the level of 8-oxo-2′-deoxyguanosine (8-OHdG), a marker of oxidative DNA damage, was significantly elevated in the brains of these mutant mice. Interestingly, this increase was age-dependent. Additionally, the activities of antioxidant enzymes, such as Cu/Zn-SOD and GPx, were enhanced in the brains of the Klotho mutant mice. Two years later, Yamamoto et al. conducted a study that further confirmed Klotho’s protective role against oxidative stress, as suggested by Nagai et al. Their findings showed that Klotho-overexpressing mice lived longer, exhibited lower levels of 8-OHdG, and had higher levels of Mn-SOD compared to controls [166].

The protective effects of Klotho have also been observed in kidney diseases. In mice with immune-mediated glomerulonephritis, increasing Klotho levels through genetic manipulation improved kidney function and reduced mitochondrial oxidative stress, including DNA fragmentation, superoxide anion generation, lipid peroxidation, and apoptosis. In humans, low Klotho levels in blood, urine, and kidney have been strongly associated with acute kidney injury, with Klotho levels correlating with resilience to kidney damage [167]. In pregnancies complicated by preeclampsia, placental mKlotho expression, measured by real-time PCR, was found to be lower compared to healthy pregnancies [47,168]. Additionally, a significant downregulation of soluble Klotho (sKlotho) was observed in the urine of women with preeclampsia compared to healthy controls [64].

The sKlotho protein, released into circulation following enzymatic cleavage of mKlotho, exhibits cytoprotective effects that extend to distant organs, including the lung. In cases of acute kidney injury, blood circulation of sKlotho is reduced, which, in turn, increases the risk of pulmonary complications [169].

In A549 lung epithelial cells exposed to hyperoxic conditions (95% O_2_) and high inorganic phosphate concentrations (3–5 mM) to induce oxidative damage to DNA and proteins, Ravikumar et al. observed an increase in cellular antioxidant capacity when sKlotho-containing conditioned media was added. In vivo, they administered sKlotho-containing conditioned media into rat peritoneum before and during hyperoxia treatment, resulting in reduced alveolar interstitial edema and oxidative damage [155]. In elderly subjects with interstitial lung abnormalities, sKlotho levels in serum were found to be lower compared to healthy individuals, suggesting that sKlotho concentrations could serve as a predictive biomarker for accelerated lung function decline in individuals with interstitial lung abnormalities [170], although not for lung cancer [171].

The brain, being highly metabolically active, is particularly vulnerable to oxidative damage, which has been linked to several neurodegenerative diseases and mild cognitive impairment [172,173]. In 2014, Semba et al. studied 70 patients, both male and female, young and old, with and without Alzheimer’s disease. They measured sKlotho concentrations in cerebrospinal fluid using an ELISA assay. The results showed that sKlotho levels were lower in females compared to males (632–801 pg/mL vs. 814–983 pg/mL, *p* = 0.002), in patients with Alzheimer’s disease compared to those without (603–725 pg/mL vs. 705–828 pg/mL, *p* = 0.02), and in older adults compared to younger adults (658–874 pg/mL vs. 884–1100 pg/mL, *p* = 0.005) [174]. In a longitudinal study involving 1453 adults enrolled from 1998 to 2000, with a 3-year follow-up from 2001 to 2003, higher plasma sKlotho levels were found to be independently associated with better cognitive performance and less cognitive decline [175].

A study by Lim et al. was the first to characterize the tissue expression of mKlotho in humans. Immunohistochemistry revealed mKlotho expression in kidney, arterial, epithelial, endocrine, reproductive, and neuronal tissues, but not in the liver [176].

However, sKlotho also exerts a protective role in the liver by influencing its energy metabolism. Rao et al. investigated the effects of 5 weeks of peripheral administration of sKlotho in high-fat diet-induced obese mice. Their findings showed that sKlotho administration reduced lipid accumulation in both the liver and adipose tissue compared to controls. Though the exact mechanisms are not fully understood, this study highlights sKlotho’s potential role in regulating energy metabolism in adipose tissue [177], similar to the role of other hormones such as oxytocin [178].

The discovery that Klotho is also expressed in peripheral arterioles has led to the hypothesis that it may be linked to cardiovascular diseases [176]. Endothelial dysfunction is a key contributor to the development and progression of CVDs [179,180]. Nitric oxide (NO) plays a crucial role in maintaining vascular tone and endothelial function [181]. In endothelial cells, NO is synthesized from L-arginine by the enzyme nitric oxide synthase (NOS). Saito et al. first provided evidence that Klotho helps preserve endothelial function by stimulating NO production [182]. A subsequent study using animal models confirmed that Klotho deficiency in mice led to endothelial dysfunction, possibly due to reduced NO production [183]. In human aortic endothelial cells, endogenous Klotho promoted NO production following administration of FGF23 [184]. Reduced NO production, in turn, leads to compromised endothelial function, which is indicative of endothelial cell senescence.

Several studies suggest that Klotho’s role in endothelial cell senescence in CVDs is mediated by Sirtuin (SIRT1) [185]. SIRT1, highly expressed in endothelial cells, is involved in regulating aging-related processes such as endothelial cell senescence, inflammation, and apoptosis [186,187]. Research by Gao et al. demonstrated that Klotho-deficient mice with arterial stiffness and hypertension had reduced serum Klotho levels (approximately 45% lower) and that activating SIRT1 could reverse some of these effects. Their study also showed that Klotho deficiency led to decreased activity of endothelial nitric oxide synthase and AMP-activated protein kinase α (AMPKα) in aortic tissues [188]. In a similar vein, other studies have shown that supplementing with ghrelin, berberine, or exogenous Klotho can revert senescence in aging mice [189,190].

### 6.2. Klotho, Placenta and Preeclampsia

Klotho plays a crucial protective role in reproductive development. In pregnant mice, exposure to perfluorooctane sulfonate (PFOS) reduced Klotho expression in the placenta, increasing the risk of birth defects in fetuses [191]. Klotho knockout pigs failed to carry pregnancy to term [192]. Genetic studies in humans suggest that Klotho gene deficiency is associated with vascular calcification, premature bone disease, altered angiogenesis, hypertension, and left ventricular hypertrophy—hallmarks of premature vascular aging [193]. Research focusing on pregnancy has uncovered associations between Klotho and various obstetrical pathologies, particularly those related to placental aging and fetal growth. Recent studies have shown that pregnant women exhibit higher median plasma concentrations of α-Klotho compared to non-pregnant women, with levels increasing as pregnancy progresses. While the exact source of α-Klotho during pregnancy remains unclear, it is hypothesized that changes in maternal or placental production may contribute to its altered expression. It is believed that gene expression and protein synthesis predominantly occur in the placenta, rather than in the mother or fetus [194]. The synthesis of α-Klotho in the placenta is particularly noteworthy, as it plays key roles in angiogenesis [195], adipogenesis [196], calcium [197] and glucose metabolism [196], antioxidant effects [166], insulin signaling [196], and phosphate metabolism [198]. For instance, Ohata et al. reported that serum samples from the umbilical vein of newborns contained higher concentrations of α-Klotho compared to 4-day-old infants, mothers, and adult volunteers [194]. Similarly, Godang et al. found higher α-Klotho concentrations in neonatal umbilical cord plasma than in maternal plasma at 32–34 weeks of gestation, confirming its expression in the syncytiotrophoblast, the epithelial layer covering the highly vascular embryonic villi of the placenta, which is in direct contact with maternal blood [199]. Immunolocalization studies have shown that α-Klotho is predominantly located in the syncytiotrophoblast [194] along with ADAM17, a metallopeptidase acting as a TNFα-converting enzyme [200] and fibroblast growth factor receptor-1 (FGFR1) [201]. It is proposed that placental α-Klotho acts as a co-receptor for FGFR1 in the syncytiotrophoblast, and the complex of fibroblast growth factor 23 (FGF23), α-Klotho, and FGFR1 may contribute to vitamin D, phosphate, and calcium metabolism [201]. Furthermore, the shedding of α-Klotho by ADAM17 could release the protein into maternal circulation, potentially explaining the elevated plasma concentrations of α-Klotho during pregnancy [200]. Interestingly, ADAM17 levels are higher in placentas from pregnancies complicated by preeclampsia compared to those from normal pregnancies [202].

Placental development is a tightly regulated process during pregnancy, ensuring fetal growth by providing oxygen and nutrients while removing waste products such as carbon dioxide [37,203,204,205].

The placenta also acts as an endocrine organ, influencing both maternal and fetal metabolism. The median maternal plasma concentration of α-Klotho has been found to be lower in women with fetal growth retardation compared to those with normal-weight deliveries [206]. Shao et al. observed elevated α-Klotho mRNA and protein levels in the placentas of women with macrosomia, regardless of gestational diabetes, compared to those who delivered normal-weight babies [207]. Additionally, serum levels of α-Klotho in both maternal and umbilical cord blood were positively correlated with fetal birth weight [208], suggesting that α-Klotho plays a metabolic role in fetal growth. In contrast, lower maternal plasma levels of α-Klotho are observed in women who deliver small-for-gestational-age babies, independent of preeclampsia status [206]. The connection between Klotho and preeclampsia has been extensively studied, and Klotho is being considered as a potential marker for diagnosis and treatment [47,209]. However, the underlying mechanisms remain unclear. One possibility lies in the aging of the placenta. In normal pregnancies, oxidative stress during later stages is associated with an increase in syncytial nodes, which exhibit signs of oxidative damage, a characteristic of normal placental aging [210,211]. Syncytial node formation is further elevated in preeclampsia, suggesting premature placental aging. Despite expectations that α-Klotho, an anti-aging protein, would be linked to placental aging phenomena such as accelerated villous maturation, this association was not confirmed. Instead, maternal plasma levels of α-Klotho were higher in preeclampsia, despite the absence of accelerated villous maturation. This discrepancy has been explained by the finding that low maternal α-Klotho levels could activate the p53/p21 pathways, increasing cellular senescence and contributing to accelerated placental villous maturation [212,213]. In the context of placental oxidative stress, the Nrf2/ARE antioxidant signaling pathway plays a crucial role in maintaining oxidative balance and has been implicated in preeclampsia [9,214]. Klotho has been shown to modulate the Nrf2/ARE pathway [215], as it activates Nrf2 in podocytes to alleviate diabetic nephropathy [216]. Thus, Klotho may influence preeclampsia pathogenesis by modulating the Nrf2/ARE pathway. Studies also show that Klotho expression is downregulated in hypoxia-treated trophoblasts and preeclamptic rat tissues, suggesting a correlation between Klotho levels and disease progression [217]. Klotho is thought to exert its antioxidant effects through inhibition of the insulin/insulin-like growth factor-1 (IGF-1) pathway and activation of FoxO proteins. The next section will focus on the inhibition of the insulin/IGF-1 pathway and the activation of FoxO proteins.

### 6.3. Klotho Regulates Mytho Gene Expression via FoxO Proteins

The insulin/insulin-like growth factor-1 (IGF-1) signaling pathway plays a critical role in determining longevity, as demonstrated by various studies in organisms such as the nematode *Caenorhabditis elegans*, the fruit fly *Drosophila melanogaster*, and several rodent models [218].

This pathway involves IGF-1, its receptor (IGF-1R), and various binding proteins.

Insulin-like growth factor-1 (IGF-1) was first discovered in the late 1950s by researchers studying the mechanisms of growth hormone (GH, or somatotropin) in animal models [219]. The IGF-1 gene, located on chromosome 12, spans at least 90 kb of DNA and contains six exons [220]. Several IGF-1 mRNA variants have been identified, primarily due to alternative transcription initiation and splicing events [221]. These mRNAs differ mainly in their 5′ and 3′ untranslated regions [222]. The IGF-1 peptide is composed of 70 amino acids (~7.65 kDa) [223] and is released by the liver under the control of growth hormone [224].

The gene encoding the human IGF-1R is located on chromosome 15 and is ubiquitously expressed [225]. The receptor is a tetramer consisting of two extracellular α-chains and two intracellular β-chains [226]. The β-chains contain an intracellular tyrosine kinase domain, which undergoes autophosphorylation upon IGF-1 binding to IGF-1R [227]. This autophosphorylation activates the receptor, triggering the phosphorylation of several cellular proteins, including members of the IRS family [228]. Phosphorylated IRS proteins can activate phosphatidylinositol 3-kinase (PI3K) and Akt [229]. Akt is a serine/threonine kinase that has been extensively studied due to its involvement in various cellular processes including carcinogenesis [230,231]. One of the main targets of Akt activity is FoxO proteins [232]. Upon phosphorylation, FoxO proteins are retained in the cytoplasm and are unable to translocate to the nucleus to activate the transcription of their target genes [232].

FoxO proteins belong to a superfamily of forkhead transcription factors. Members of this superfamily share a conserved 110 amino acid DNA-binding domain known as the “forkhead” or “winged helix” domain [233]. The FoxO factors are homologous to DAF-16, a gene in *Caenorhabditis elegans* that plays a key role in regulating the aging process [234]. In mammals, there are four FoxO members: FoxO1, FoxO3, FoxO4, and FoxO6 [235]. In humans, FoxO1, FoxO3, and FoxO4 are ubiquitously expressed, including in placental tissue [236,237]. FoxO3 is particularly abundant in tissues such as the brain, heart, kidney, and spleen [238,239,240,241], while FoxO6 mRNA is predominantly expressed in the developing and adult brain, suggesting a role in the nervous system [242]. Several studies have shown that Klotho can inhibit the insulin/insulin-like growth factor-1 (IGF-1) signaling pathway [166,243]. By blocking the PI3K/Akt-mediated phosphorylation, FoxO factors translocate to the nucleus, where they promote the transcription of genes involved in metabolism, cell cycle regulation, apoptosis, stress resistance, DNA repair, and antioxidant defense. Key antioxidant genes such as catalase (CAT) and superoxide dismutase (SOD2) are among these targets [243,244,245]. Studies in animal models and human cell cultures have shown that Klotho overexpression or the addition of sKlotho to culture media increases Mn-SOD expression and reduces FoxO phosphorylation levels [166,246]. In the brain of senescence-accelerated mice (SAMP8), inhibition of the Akt/FoxO1 pathway led to increased expression of MnSOD and catalase, enhancing DNA repair [247]. In aged SAMP8 mice, Klotho deficiency was linked to FoxO1 inactivation and a reduction in antioxidant enzyme levels [247]. Further studies using Klotho +/− BALB/c mice demonstrated that administration of sKlotho dramatically reduced the level of 8-OHdG in urine, suggesting improved DNA repair activity through FoxO3a binding to the MnSOD promoter. This effect was dependent on Klotho-mediated inhibition of PI3K/Akt signaling [243].

**Figure 1:**
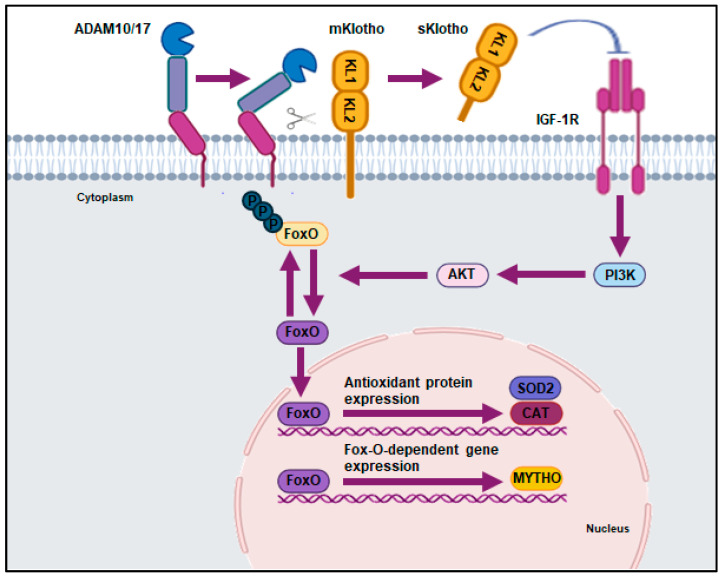
Soluble form of Klotho (sKlotho) and antioxidant mechanisms. Once the membrane-anchored secretases AD-AM10/17 cleaves the membrane form of Klotho (mKlotho), the extracellular domain is released into the extracellular space. The extracellular domain represents the soluble form of Klotho (sKlotho) which, via peripheral circulation, reaches the cell target and inhibits the insulin/IGF-1/PI3K/Akt/FoxO pathway. Therefore, the expression of genes related to antioxidant defense (such as superoxide dismutase (SOD2) and catalase (CAT)) is promoted and the oxidative stress decreases. Similarly, FoxO proteins induce the transcription of the Mytho gene, a regulator of autophagy and skeletal muscle integrity.

Recently, Leduc-Gaudet and colleagues identified a new FoxO-dependent gene, Mytho (Macroautophagy and YouTH Optimizer) [248]. Though Mytho’s exact role is still being understood, experimental data suggest that it acts as a regulator of autophagy and skeletal muscle integrity. In mice with skeletal muscle atrophy, Mytho protein was overexpressed in muscle tissue, while silencing the Mytho gene resulted in increased muscle mass. Prolonged silencing led to severe myopathy. sKlotho may regulate Mytho expression through FoxO proteins (Figure 1). The prolonged silencing of Mytho may explain the musculoskeletal frailty observed in elderly individuals and women with preeclampsia.

## 7. Discussion

Pregnancy represents a unique biological state in a woman’s life and may serve as either a senescence or rejuvenation event for the body. Senescence plays a crucial role in various pregnancy-related processes, including syncytiotrophoblast formation, placental implantation, embryonic development, and delivery [249]. During pregnancy, many metabolic changes occur that contribute to aging, such as alterations in DNA methylation, increased inflammation, oxidative stress, and telomere shortening [250,251,252]. However, after a healthy pregnancy, women experience a period of rejuvenation and repair in the weeks and months following delivery [252]. It remains uncertain whether pregnancy accelerates, slows, or prolongs the aging process. The answer likely lies in the complexity of these events, which are influenced by a woman’s biological and genetic makeup, the type of pregnancy, and the postpartum timeline. Notably, pregnancy complications such as preeclampsia are associated with an increased risk of cardiovascular and neurovascular events in later life. These events tend to occur earlier in life than in women with uncomplicated pregnancies and are often preceded by a prolonged asymptomatic period.

The connection between preeclampsia and aging is supported by research on the Klotho gene, an anti-aging protein that appears to play a pivotal role during the intrauterine period and may influence long-term health outcomes. Current insights into the link between Klotho and intrauterine metabolism are promising, but need further exploration.

## 8. Conclusions

This review highlights the complex relationship between preeclampsia and aging, emphasizing shared biological and pathological features such as oxidative stress, inflammation, and cellular senescence. The Klotho gene emerges as a key regulator in these processes, exhibiting protective roles across various organ systems by modulating oxidative stress, inflammation, and metabolic pathways. Its involvement in placental function and pregnancy outcomes further underscores its importance in the pathogenesis of preeclampsia.

Future research should aim to elucidate the molecular mechanisms underlying Klotho’s actions and explore its potential as a biomarker for early diagnosis and therapeutic interventions. Furthermore, investigating the connection between Klotho expression and the recently identified Mytho gene offers a promising direction for understanding the broader implications of senescence and metabolic regulation in both pregnancy and aging.

## 9. Future Directions

The placenta plays a central role in preeclampsia, providing a unique lens through which to examine aging processes. Syncytial knots and other markers of premature placental aging are more prevalent in preeclampsia, reflecting oxidative stress and cellular senescence. The relationship between placental Klotho expression and fetal outcomes highlights its importance in intrauterine programming and long-term health. Notably, elevated levels of α-Klotho in maternal plasma during pregnancy may act as a compensatory response to counteract oxidative stress. However, its dysregulation in preeclampsia suggests potential diagnostic and therapeutic applications. Olivieri et al. demonstrated that long-term cultured HUVECs, a model of replicative cell senescence, release exosomes that induce a pro-senescent phenotype in co-cultured target cells [253]. Building on this, we propose the establishment of a co-culture system of long-term cultured HUVECs as a model for preeclampsia, with kidney or hepatic cells. Characterizing the exosomal content—such as miRNAs and proteins—could provide valuable insights into the potential role of Klotho as a clinical biomarker. This could open up new therapeutic avenues for managing pregnancy-related conditions and preventing fetal complications.

This review outlines several promising avenues for future research:

1. Mechanistic Studies: Further investigation into Klotho’s interactions with the Nrf2/ARE pathway, FoxO proteins, and epigenetic regulators could shed light on its protective mechanisms in both preeclampsia and aging.
2. Biomarker Development: Exploring Klotho as a biomarker for the early diagnosis of preeclampsia and age-related diseases is crucial. Profiling circulating Klotho levels, along with associated epigenetic markers and miRNAs, could enhance predictive accuracy.
3. Therapeutic Interventions: Developing strategies to upregulate Klotho expression or mimic its effects through pharmacological agents or gene therapy could revolutionize the management of preeclampsia and age-related disorders.

## Data Availability

No new data were created or analyzed in this study. Data sharing is not applicable to this article.
